# Themes Across New Directions in Community Engagement

**DOI:** 10.3390/ijerph16193724

**Published:** 2019-10-03

**Authors:** Shannon M. Cruz

**Affiliations:** Department of Communication Arts & Sciences, The Pennsylvania State University, University Park, PA 16802, USA; shannon.cruz@psu.edu

**Keywords:** community engagement, community involvement, Superfund

## Abstract

The articles in this special issue on New Directions in Environmental Communication in the *International Journal of Environmental Research and Public Health* present new research and perspectives on engaging communities impacted by Superfund sites—the hazardous waste sites that have been identified by the U.S. Environmental Protection Agency (EPA) as needing cleanup. In particular, these articles focus on the community engagement cores (CECs) that work with affected communities as part the Superfund Research Program at the National Institute of Environmental Health Sciences (NIEHS). The purpose of this closing article is to highlight important themes evident across the eight articles in the special issue. When considered together, the findings reveal important lessons learned about community engagement and environmental communication, but also reveal that much more remains to be known. Recommendations are made for how these teams can continue to practice, reflect on, and research community engagement in ways that build toward a better understanding and implementation of best practices.

Dearing began this special issue of the *International Journal of Environmental Research and Public Health* with a call for social and behavioral scholars to help address the *clean-up gap* ([1], p. 2), which refers to the widening gap between the number of hazardous waste sites that can be remediated and the number that have been documented. Dearing argues that technical innovations are vital to remediation efforts, but what is most needed to narrow the clean-up gap is a better understanding of the social factors involved and better sharing of best practices. Accordingly, this special issue of *IJERPH* features research and commentary from some of the academic and institutional stakeholders that support the U.S. Environmental Protection Agency’s (EPA) Superfund program and the Superfund Research Program (SRP) at the National Institute of Environmental Health Sciences (NIEHS). 

This paper closes out the special issue by identifying lessons learned from a reflection on this body of work. The goal of this analysis is to provide a preliminary response to Dearing’s call by assessing what these articles reveal about some of the ongoing community engagement efforts at Superfund sites and other hazardous waste sites. The paper begins with background on community engagement, continues with a summary and analysis of the articles in the special issue, and finishes with a discussion about future research and practices that would help SRP teams continue to serve communities better.

## 1. Background

Since the Superfund program and SRP were established in the U.S. in the 1980s, they have had an important role in involving citizens in the remediation of Superfund sites affecting their communities. In the SRP, this role is accomplished primarily by requiring each university-based center to have a Community Engagement Core (CEC). The CECs seek to “enhance knowledge exchange and to support the needs of communities impacted by hazardous-waste sites” [2] (para. 4) through the process of community engagement, which the SRP defines as “bidirectional interaction and communication between community stakeholders and its Centers” [3] (p. 2). As will be discussed in greater detail later in this paper, CEC activities vary widely, ranging from relatively low-engagement activities such as outreach and consultation [4,5] to high-engagement activities such as community-based participatory research [6].

In the EPA, this role is accomplished by community involvement staff, who engage in “dialogue and collaboration with community members” ([7], para. 1). The EPA is required to document community involvement in the Record of Decision (ROD) for a Superfund site and to provide a public meeting and comment period [8], though the efforts of community involvement staff also “often go well beyond” what is legally required ([9], p. 168). Activities include informing the public about the hazard and cleanup process, addressing environmental justice concerns, training community involvement staff in best practices, and offering technical assistance grants to communities, among others [10].

Although community engagement is not part of the physical process of cleaning up a hazardous waste site, it is undoubtedly vital to remediation efforts [10]. As Senier et al. [11] put it, “remediation ought to restore a community’s sense of wholeness, safety, and integrity, as well as restoring clean air, soil, and water to their environs” (p. 4661). In other words, the remediation process is incomplete if it does not address both physical and social aspects of a contaminated site. Previous research indicates that when it is executed well, community involvement at Superfund sites can provide a number of benefits, including better citizen knowledge about the remediation process; greater satisfaction with the EPA’s cleanup efforts [9]; improved quality, credibility, and acceptance of proposed solutions [11]; and faster construction completion times [12]. More generally, community engagement has also been found to have a positive effect on health behaviors, health outcomes, self-efficacy, and perceived social support among community members [13].

To date, the efforts of EPA staff and SRP centers have contributed to the successful remediation of over 400 sites on the Superfund National Priorities List (NPL) and to the completion of construction projects on many other NPL sites since Superfund’s inception [14]. However, substantial challenges for community engagement at Superfund sites remain. According to a report by the Government Accountability Office (GAO) [15], around 39 million people lived within three miles of a nonfederal NPL site as of 2013—around 13% of the U.S. population at the time. In states with a larger number of NPL sites, the proportion was much higher, including as many as 29% of New York residents and 50% of New Jersey residents. Many of these residents are still awaiting remediation, and they will depend on the efforts of EPA and CEC personnel to ensure that remediation conveys social as well as environmental and health benefits.

In addition, the EPA is currently facing growing financial barriers to beginning and completing remediation of Superfund sites. According to the same GAO report [15], although new sites continue to be added to the NPL each year, government appropriations for the Superfund program have steadily declined. As a result, the total amount the EPA has been able to spend on remediation has declined, the rate of project completion has slowed, and initiation of some new remediation activities has been delayed. If these trends continue, the rate at which growth in NPL sites outpaces remediation can only increase. As it does, community engagement efforts are likely to play an increasingly important role in addressing this clean-up gap [1]. Although EPA community involvement staff and SRP teams may not be able to change the pace of remediation directly, they can work to make community engagement more effective and efficient, and may even be able to begin addressing social and financial impacts of contamination before remediation occurs [11].

Given both the importance of community involvement to remediation efforts and the scope of involvement activities that will be required to address all remaining NPL sites, it is not surprising that both agency staff and academic researchers have endeavored to study and improve community engagement processes. For example, the EPA has acted to evaluate and update its community involvement efforts in response to the Government Performance and Results Act [9]. Anecdotal evidence [16] indicates that these efforts have been beneficial, and other research finds that the pace of cleanup of Superfund sites has become substantially faster than it was when the program was first established [8], providing an indirect indicator of improvements in community involvement. Efforts to promote environmental justice in the cleanup process have also had a positive impact—although minority communities experienced longer cleanups when Superfund was first established, that difference has disappeared over time [12].

These improvements are encouraging, but there is still ample room to make these programs more successful. Previous research on community involvement in Superfund, for example, has found that neighborhoods participate unevenly in the community involvement process. Those in poorer neighborhoods are more likely to be active, whereas racial and ethnic minorities and non-homeowners may be less active ([8], though cf. [16]). Involvement can also come with drawbacks. Some authors [8,17,18] have found that higher levels of citizen participation are associated with longer remediation processes, and Petrie [8] also found that they were associated with a greater likelihood that capping will be used to contain a site (which she described as a sub-optimal solution for managing the hazard) (though cf. [19]). It can also be challenging to determine what forms of community engagement have tangible impacts [11] or are cost-effective [20].

To some extent, EPA community involvement staff and SRP CEC teams can continue to improve by examining the existing literature on community engagement. It has been the subject of systematic literature reviews [13,21,22], a feature of special issues in journals such as the *International Journal of Hygiene and Environmental Health* [23,24] and *Translational Behavioral Medicine* [25], and the topic of numerous research projects from diverse fields and methodological perspectives [17,26,27]. Elsewhere in the literature, practitioners can also find resources on related topics such as public participation [9], community participation [13], and community-based participatory research and civic science [28,29].

However, although EPA staff and CEC teams can and should refer to this literature, it is also important to note that communities impacted by Superfund sites may in some ways be different from communities deliberating about other environmental issues. As Stephan [16] points out: “Unlike the siting of waste facilities, toxic waste sites are problems that already exist in people’s backyards. Because the problems are already there, people who want to influence cleanups are required to go beyond a simple negative reaction and think through in more detail what they want and why” (p. 665). In other words, because Superfund sites place different demands on community members, research on community engagement broadly may not always generalize to the contexts in which EPA community involvement staff and SRP CEC teams work.

The only way to improve understanding of the unique contexts that Superfund sites create is to devote more energy to studying and reflecting on community engagement at these sites, particularly on the experiences of CEC teams. Although there is some work specific to Superfund [9,24,30,31,32,33], the body of research on community engagement processes specific to these sites is more limited, and analyses of the work of SRP CECs is more limited still. Published work on CECs tends to report on efforts of a specific team [6,11,34] without drawing connections between the work of different CECs. Even a recent article reflecting on 25 years of SRP achievements [35] makes very little mention of community engagement. This special issue thus presents a timely opportunity to build on lessons learned so far and consider what more remains to be studied.

## 2. Current Research and Perspectives

The articles in this special issue of *IJERPH* represent a sample of perspectives and research on community engagement at Superfund sites. The work presented is quite diverse, but it can be divided into a few broad categories.

First, two of the articles provide agency perspectives on Superfund and the SRP. Trottier et al. [3] focus on the importance of community engagement and research translation to the NIEHS and the role of CECs and research translation cores (RTCs) in SRP centers. The authors emphasize that both are important components of the SRP mission and describe some of the ways that SRP teams have engaged communities effectively, including developing tools to improve citizen knowledge and health literacy, adapting communication strategies to communities’ culture-specific needs, and forging partnerships that connect community members to research being conducted in SRP centers. On the other hand, Zaragoza [10] focuses on the EPA’s community involvement efforts at Superfund sites. He explains that not only is community involvement one of the core goals of the Superfund program, it is also vital to reaching the program’s other goals: protecting human and environmental health, holding responsible parties accountable, and enabling communities to resume use of previously contaminated sites. He provides a review of the methods that the EPA uses to involve communities affected by Superfund sites and reflects on the challenges that these efforts may face.

Two other articles also present broad reflections on community engagement, but from the perspective of SRP teams. First, Cordner et al. [28] propose a typology of research that combines social science and environmental health. They suggest that research at the nexus of these two disciplines can be divided into; “1) environmental health science influenced by social science; 2) social science studies of environmental health; and 3) social science-environmental health collaborations” (p. 2). The first category describes cases when environmental health researchers take social science factors into account, such as often occurs on SRP CEC teams; the second category refers to cases when social scientists conduct research on environmental health issues, such as a case study of a contaminated community; and the final category refers to cases where social scientists and environmental health scientists work collaboratively on research. Cordner et al. argue that this third form of research is often the most challenging, but can also be the most rewarding and contribute the greatest improvements to human and environmental health. Cox et al. [36], in contrast, develop a set of propositions for when CECs can be more successful by engaging with the regulatory community rather than directly with citizens. They suggest that when a community has issue fatigue (i.e., they have heard about an issue for a long time, and are no longer eager to be involved), is economically or socially reliant on the party responsible for the contamination, and is already being engaged by other institutional stakeholders, CECs can provide more benefit to a community by assisting agencies with ongoing engagement efforts than by working independently.

Finally, the four remaining papers present research conducted by different SRP CECs. First, Rogers et al. [4] describe the creation of a website for sharing information on arsenic risks. The authors consulted with experts to develop a model of arsenic exposure, used this model in the creation of a comprehensive website about arsenic, and compared the expert model to community understanding of arsenic risk. Their results revealed that the website had a wide reach and was successful in impacting visitors’ knowledge and behavioral intentions, but community members still had a less complex understanding of arsenic risk than experts. Second, Zwickle et al. [37] present an analysis of the impact of dioxin contamination and remediation on property values. These authors found that dioxin contamination had a negative impact on property values that remediation did not necessarily remove, though remediation at least did not cause more economic damage. Third, Zhuang et al. [38] assess the framing of dioxin contamination and remediation efforts in local and national news coverage. These authors found that newspapers differed in their coverage of environmental contamination, sometimes focusing on the risks and sometimes on the remediation process. Community participation was only mentioned rarely by the articles reviewed and was typically portrayed in a negative light when it was. Finally, Middleton et al. [5] describe their experiences and challenges in community engagement with the Yurok Tribe in California. These authors explain that they have faced several challenges so far, including difficulties in getting non-CEC members of the SRP center to participate in engagement and knowledge exchange, difficulties in finding the best avenues for community outreach, misunderstandings about realistic outcomes of engagement efforts, and disconnects between what the community hoped to get out of the experience and what the SRP center has been able to provide. They note that the challenges have been difficult to overcome, but reflection on the experience has also given center members a new perspective on engagement.

## 3. Themes Across Current Research and Perspectives

At first, it is challenging to see how these papers all connect. However, they do make certain things clear about SRP CECs and the state of community engagement in general.

**Theme** **1:** *Community engagement is widely valued, but teams may still need to work to make sure that the importance of these efforts is understood by everyone on an SRP team*.

One thing made clear by the articles in this special issue of *IJERPH* is that community engagement and research translation are increasingly valued by the agencies and universities that work to study and remediate hazardous waste sites. This commitment to working with communities is a clear priority of the Superfund program [10] and of the SRP [3].

To reiterate, Zaragoza [10] explains that the EPA views community involvement to be “an integral part of the Superfund cleanup process” (p. 10). Community input and buy-in make clean-up efforts more successful, providing better protections for human and environmental health; grassroots efforts can encourage new legislation and put pressure on polluters to take responsibility for clean-up costs; and community participation makes it more likely that hazardous waste sites will eventually return to productive use. This commitment to community engagement is reflected in both formal policies and in the efforts of community involvement staff.

Similarly, Trottier et al. [3] explain that the NIEHS views community engagement conducted by SRP centers to be “integral in communicating, promoting, and disseminating research” (p. 5). Along with research translation, community engagement activities are central to prevention and intervention activities that reduce community members’ exposure to hazardous substances and ensure that “communities have an opportunity to benefit from the research” (p. 5) that is conducted by SRP centers. The commitment to community engagement is reflected in the 2010 decision to require all new centers to incorporate CECs [39], and in the ongoing efforts of CECs around the country.

However, the institutional commitments to community engagement do not necessarily imply that its value will be immediately understood by all parties. Middleton et al. [5] explain that one of the challenges faced by their CEC, for example, was that other members of the SRP center were not always eager to participate in community engagement activities. When they set up training sessions that brought university researchers and community members together to learn about the Yurok Tribe and research conducted by the Yurok Tribe Environmental Program, attendance among researchers was low, and some that did attend “wondered how it was relevant to their lab-based research” (p. 17). This experience is also not unique to this particular SRP center. Senier et al. [11] explain that engagement can often be challenging “given that many biomedical and engineering scientists are not trained to recognize and address social problems plaguing these communities” (p. 4656).

These experiences emphasize that it cannot be taken for granted that all SRP personnel will necessarily understand the value of engagement activities. Although both the EPA and NIEHS have come a long way when it comes to appreciating the value of community involvement, CECs may still need to take steps to explain this value to members of their SRPs. For example, Middleton et al. [5] found that drawing more explicit connections between engagement and research helped other members of the SRP center better understand the value of community engagement activities.

**Theme** **2:** *Community engagement is not very well defined, and people interpret this mandate very differently. Many teams would benefit from defining their goals more carefully*.

Although people across the EPA, NIEHS, and SRP tend to agree that community engagement is important, they do not necessarily agree about what community engagement means or what its goals are. Indeed, even though this special issue of *IJERPH* highlights only a sample of the community engagement efforts taking place at CECs around the U.S., the task of identifying common themes across the articles was still challenging. Based on the work presented here, community engagement may include the study of the communication process in communities [38], the study of other social issues affecting a community [37], the practice of communicating with community members [4], the process of engaging with institutional stakeholders in a community [36], and reflection on the community engagement process itself [5].

On the one hand, the diversity of research and perspectives is encouraging. It illustrates that CEC teams think about community engagement creatively and are well aware of the breadth and complexity of social factors that may affect the communities impacted by Superfund sites. Teams are also clearly willing to adapt their practices to a specific community context rather than adhering to a generic set of engagement practices. The focus on both researching and practicing community engagement also illustrates that teams are aware of the need to not only serve communities, but to reflect on best practices and work to improve them.

However, to the extent that this diversity stems from a lack of clarity about what community engagement is and should achieve, it is likely to be responsible for a range of problems. For one, SRP teams may define community engagement very differently than the EPA does. For the EPA, community involvement is defined narrowly and explicitly [10], in part because certain activities are federally mandated. Community engagement cores, on the other hand, are afforded comparative flexibility in the activities that they can pursue. This flexibility allows CEC teams to address a wider scope of concerns than EPA community involvement staff can, but it also means that CECs may need to take extra care not to disrupt activities that are already ongoing at Superfund sites [10,35]. As Zaragoza [10] emphasizes, “there is a widely held expectation that government agencies coordinate amongst themselves as communities should not be expected to distinguish messages from different agencies” (p. 9). Likewise, CEC teams need to make sure that their efforts are complementary to EPA efforts, which requires a clear understanding of what engagement will involve and seek to accomplish.

The lack of clarity also makes it challenging to determine whether or not engagement activities have been successful. This problem was identified 20 years ago by Chess and Purcell [21], who noted that defining the success of community engagement was “problematic because of the diversity of perspectives about the goals of public participation” (p. 2685). According to their review, success of community engagement has been defined based on the outcomes (such as knowledge gain or decision quality), the process (such as fairness), or a combination of the two. They also find that satisfaction with process does not always imply satisfaction with outcomes, emphasizing that it is important to consider carefully which is the priority and whether or not both are being addressed. Though what Chess and Purcell define as public participation may be somewhat different from community engagement as envisioned by the SRP, their point is still well taken. SRP CECs need to establish early on whether they are prioritizing outcomes or processes and what specific goals this includes (see [33] for an example). If these aspects of community engagement are not defined clearly, then it is very challenging to determine which activities are appropriate, how they should be evaluated, and whether or not they have been successful.

If goals are not defined well and discussed explicitly, then CECs are also likely to create problems with follow-through from a community perspective. For example, one of the challenges that Middleton et al. [5] reported experiencing was a misunderstanding about project outcomes. They explain that when a group of undergraduates participating in the International Genetically Engineered Machine competition was brought to meet with the Yurok tribe, they spoke with tribe members about developing a sensor to detect soil contaminants. The students’ goal was only to develop a proof-of-concept for the sensor, but the tribe members were given the impression that the sensor itself was going to be developed. This misunderstanding may have occurred in part because the goals of this activity were not clarified in advance or communicated explicitly with community members. Several authors have noted that community members are often frustrated by a lack of follow-through on the part of agencies and academic researchers [11,24], and CEC teams need to be careful not to commit the same mistakes. Middleton et al. [5] put it well when they state that their experience “highlights the importance of communicating realistic research and development timelines as well as the responsibility that researchers have in making efforts to ensure that community-engaged work is continued beyond short-term projects” (p. 18). Having careful conversations about what engagement activities will best serve a community and why would help CECs maintain goodwill and avoid unintended consequences that may occur when goals are not defined or communicated sufficiently.

In sum, all of the engagement activities featured in the special issue are valuable, but they also highlight a need to have more conversations about the goals of community engagement and research translation, the desired outcomes, and how to respect community member needs by providing meaningful results and deliverables, rather than ‘checking off a box’ that engagement has been done. More sharing about all forms of community engagement within NIEHS would be helpful, and communication between scholars who see community engagement in different ways would help teams identify ways to grow their community engagement and research translation efforts (e.g., from practicing communication to studying communication and vice versa). Building an understanding of best practices that can easily be diffused [1] will be impossible if teams do not have a clear understanding of what they are hoping to achieve and how those goals fit in to the overall efforts of the SRP.

**Theme** **3:** *Superfund sites are incredibly diverse, with comparable diversity in community contexts. No single set of strategies will be sufficient for success*.

Previous research on community engagement makes it clear that no single strategy will be successful in all situations. Chess and Purcell [21], for example, found that public hearings, workshops, and community advisory groups could all be successful in some situations, but “the history of the issue, the context in which the participation takes place, the expertise of those planning the effort, and the agency commitment may all have an impact on a particular program’s success or failure” (p. 2690). Likewise, Fiorino [22], in his comprehensive review of research on public engagement, concluded that “there are no easy answers…the literature is long on definitions of the problem but short on practical institutional solutions” (p. 523).

Part of the reason why the research in this special issue is so diverse is for the same reasons. Rather than relying on a one-size-fits-all strategy for community engagement, CEC teams have needed to adapt to the local context and needs of the community in which they are working to be successful [36]. In some communities, a wide range of hazards are the focus [5], whereas others may be impacted mainly by a single chemical or group of chemicals [4,37,38]. Some communities may need technical assistance [10] or help in understanding expert perspectives on a risk [4], whereas others may already have heard a lot about the risk [36] or include experts who are well versed in the hazards the community is facing [5]. And although many communities are likely to be frustrated and angry with the parties responsible for contamination [5,28], others may be defensive or see remediation efforts as an unnecessary imposition [36]. Clearly, different communities will want and need very different things from a CEC team hoping to engage them.

This reality has probably also contributed to problems with defining and setting clear goals for community engagement efforts, as discussed in the previous section. In particular, recommendations that researchers give about engagement are clearly shaped by their experiences with specific communities. This point is emphasized when considering the article by Cordner et al. [28] and Cox et al. [36] side by side. On the one hand, Cordner et al. [28] describe community engagement as a form of activism, where members of a CEC help to empower community members to confront polluters and advocate for themselves. These authors describe their focus as mainly on “marginalized and underrepresented groups and environmental justice communities” and paint a picture of communities who are passionate about confronting government and industry in a fight for environmental health (p. 2). The image of community engagement presented by Cox et al. [36], on the other hand, is very different. They argue for a focus on institutional stakeholders instead, working more to facilitate agency efforts to engage a community. In the community in which their team worked, many members were proud of the company that was responsible for the contamination and had issue fatigue from hearing about the problem over many decades, so remediation efforts were often viewed with skepticism.

The fact that such drastically different perspectives could both emerge from experiences of working with communities impacted by Superfund sites emphasizes just how much these communities vary. Accordingly, CEC teams and SRP centers in general need to be aware of what this means for engagement efforts. For one, CEC teams cannot presume to know what type of engagement activities will be needed or what community members will want before taking time to learn about the local context. In addition, it is clear that not enough is yet known about what differences matter or how engagement activities can be effectively matched to community needs. Although Cox et al. [36] offer suggestions for when an institution-oriented approach may be appropriate, this is only one piece of guidance among many that CEC teams are likely to need in order to serve communities effectively.

**Theme** **4:** *Despite the fact that different engagement strategies will necessarily differ between CECs, there are some practices that are likely to help all teams*.

The literature on public participation and community engagement abounds with recommendations about best practices, so it is not a new idea to suggest that CECs incorporate some of them into their activities. However, as CEC personnel may not always include social scientists who are familiar with this literature, it bears reiterating some of the strategies that seem particularly relevant based on the work presented in this special issue.

First, it is important to work to understand community needs before deciding what activities to pursue. Chess and Purcell [21] in their review of public participation, conclude that agencies that want to be successful should “begin participation early and invest in advance planning” (p. 2691). Likewise, Bonham and Nathan [24] argue that “researchers who address community concerns early in their research design will be more likely to engage the community” (p. 17). As the discussion of community needs in the previous section suggests, CECs cannot presume to know in advance what a community will hope to get out of a partnership with an SRP team, and the only way to find out is to dedicate time early on in the process to speaking with community members. For example, CEC teams might consider carrying out a stakeholder analysis. Reed et al. [40] note that stakeholder analysis can be a valuable part of natural resource management for two important reasons: first, it helps “empower marginal stakeholders to influence decision-making processes” (p. 1934), so that groups who would not typically have a voice can be included; and second, it helps agencies and researchers “understand the diverse range of potentially conflicting stakeholder interests” (p. 1935), so that they can be addressed. By engaging in this process, Reed et al. argue, teams can accomplish both normative goals, such as giving people the right to participate in decisions that affect their communities, and instrumental goals, such as the diffusion of a behavioral intervention. Cultural humility training [41] may also help to supplement these efforts. Cultural humility relies on self-reflection to encourage practitioners “to relinquish the role of expert, work actively to address power imbalance in communication to create respectful and dynamic partnerships with the community, and ultimately become a student of the community” (p. 318). Practices like these would help ensure that community needs have a meaningful influence on CEC efforts and that all members of a community, particularly marginalized groups, have an opportunity to share what those needs are.

Investing more time in these early conversations would not only benefit individual CEC teams (by helping them set more relevant and realistic goals, direct their activities, and avoid missteps), it would help the SRP as a whole to improve its community engagement practices. By sharing information about how community needs differ, what communities most often want from partnerships with a university center, and whether or not these wants and needs are predictable based on other factors (such as the nature of the hazards at a Superfund site, the demographic composition of the community, or the historical context), the SRP can begin to develop better guidelines for CECs and help make them more effective and more efficient in the future. Evaluating the efforts of any one center also becomes easier when there is a framework for comparing them to other centers. For example, the International Association for Public Participation [42] has developed a Spectrum of Public Participation that evaluates the extent of public involvement in decisions. The least participation occurs when activities merely seek to *inform* the community, increase as an effort is made to *consult* the community about the decision, *involve* the community in decisions by taking their goals and concerns into account, or *collaborate* with the community throughout the decision-making process, and peak when the process seeks to *empower* the community to make their own decisions. Evaluating engagement efforts along a participation continuum like this one could help the SRP understand why different centers produce different outcomes and why goals and outcomes may not always align.

Second, it is important to establish realistic expectations on both sides. Apart from establishing what the community hopes to gain from a partnership with an SRP center, it is important to consider what CEC teams can realistically provide. On the one hand, CECs need to avoid giving community members the wrong impression about what their center can accomplish, both in terms of the basic science being conducted by the center’s project cores and in terms of the social outcomes that can be delivered by the CEC and RTC [5,11]. On the other hand, CECs also need to have a realistic idea of what they can expect from a community. Often, communities may not welcome their involvement [36], and it is not uncommon for community members to express animosity and distrust of academic researchers, particularly if the community has also come to distrust other agencies associated with remediation efforts [11]. Teams may also struggle to find people to participate in research or outreach activities “because of the scarce resources and the finite energy of leaders to marshal the involvement of their communities for a project that they do not perceive as directly benefitting them” [24], (p. 17). CEC teams cannot expect that community engagement will always be easy, and it is likely to take substantial time and effort to earn a community’s trust. Altogether, CEC teams that work to develop realistic expectations among their own members and cultivate realistic expectations among community members are more likely to be successful in their engagement efforts.

Third, after becoming well acquainted with a community’s needs and establishing realistic expectations on both sides, teams need to work with communities to clarify what their goals are [21], how those goals will be accomplished, and how the team’s success in reaching them will be evaluated. It should also be clear how these goals will fit with the goals of the EPA, other agencies, and community groups that are already active in a community. By basing these goals on a solid foundation of shared understanding, CEC teams can avoid misunderstandings and conflicts with other stakeholders in a community. Checking for consonance between goals, activities, and evaluation procedures can also identify potential problems so that they can be corrected proactively, rather than after negative consequences have already been realized.

Teams also need to keep in mind that their short-term goals for a specific project or activity may differ from their long-term goals for engagement, but both sets of goals still need to be compatible. For example, an event designed to inform the public about a risk may be considered successful if it helps reach a short-term goal of education, but this success is not particularly meaningful if it does not also contribute to long-term goals like building community capacity to participate in future decisions about the risk [43]. When the focus is on short-term outcomes, teams also risk overlooking or undervaluing long-term processes that may be even more important. In particular, cultivating mutual respect, developing an appreciation for engagement among all members of an SRP center, and building and maintaining strong relationships with community members are probably among the most vital goals for a CEC team to pursue, but they also take the most time and commitment to accomplish. If CEC teams are focused only on short-term goals, they are unlikely to recognize how their efforts contribute to (or undermine) these big picture outcomes.

Finally, if the SRP is to have a meaningful role in addressing the clean-up gap [1], there needs to be a careful balance of practice, reflection, and research on community engagement in SRP centers. On the one hand, the practice of engagement is central to many CEC teams [4]. Activities such as sharing knowledge, building health literacy, and developing community-based participatory research projects can all be important elements of successful community engagement. However, practice absent reflection and research is insufficient. Within a CEC team, reflection on ongoing engagement activities is vital for gathering anecdotal evidence about which practices have worked, which should be revised, and which should be discontinued [5]. Across CEC teams, reflection can also provide insights into how communities differ from one another [28,36] in ways that would not be clear from the perspective of a particular team. And in both cases, reflecting on the existing social science literature can provide new ideas about topics that may be relevant to a center’s goals, such as science and risk communication, numeracy, and interorganizational partnerships. Research, in turn, is vital for gathering empirical evidence about what practices work. Authors have been emphasizing for decades that more research on public participation and engagement is needed [21,22], and this remains true today, particularly in contexts specific to Superfund sites. Although reflection may help a particular CEC to improve its practices [e.g., see 33], engagement across the SRP cannot meaningfully be improved without systematic research. To reiterate, the experiences of a CEC team may change drastically from one community context to another, so passing anecdotal advice between teams working in different communities is likely to be ineffective or even counterproductive. Meaningful diffusion of best practices [1] will only be possible with a much better developed sense of community needs differ and how different needs can best be addressed, which will require more research on the community engagement process itself across the SRP. Although some authors have argued that helping and researching a community are incompatible activities [27], and it is important not to expect community members to participate in research that will not benefit them [11,24], some research activities are necessary for CEC teams to evaluate and improve engagement.

## 4. Discussion

This article has sought to identify common themes across the articles presented in this special issue of *IJERPH*. These articles are so topically diverse that identifying similarities is at first challenging, but reflecting on them does indicate a few important lessons for the SRP and CEC teams to consider. In particular, there appears to be robust agreement that community engagement is valuable, but there is quite a bit of disagreement about what engagement looks like and should accomplish. The variety of strategies may be helpful in some ways, because communities are very diverse in terms of their needs and expectations, but can also introduce several problems that CEC teams need to be aware of. Adopting a few basic practices would likely help teams avoid some of these problems while still permitting them flexibility in their engagement strategies.

Considering the four lessons identified above, it is possible to make a few recommendations about how the SRP can continue to improve its community engagement efforts and help address the clean-up gap. Activities that would be helpful include:

- *Having more conversations about what community engagement means across the SRP*. Different interpretations are valuable, but teams need to have a better understanding of the scope of community engagement across the SRP and how their interpretation fits into the bigger picture. These conversations can tackle difficult questions such as: What level of engagement should CEC teams strive for? How can CEC teams incorporate practice, reflection, and research into their activities? What are the program-wide outcome and process goals for CEC teams? Should all teams share the same goals, or will these differ by community? Having careful conversations about the meaning of community engagement can also reveal important aspects of research or practice that may previously have been unnoticed or neglected. For instance, Fiorino [22] pointed out 30 years ago that “we test techniques for communicating risk information to the public, but conduct almost no research on mechanisms for the lay public to communicate with government officials and technical experts” (p. 504). Encouraging more conversations about community engagement among centers could introduce different perspectives such as this one, revealing new opportunities for research and practice.
- *Developing better guidance for new (and even existing) teams*. The requirement for all SRP centers to include CECs is fairly new, so many teams are still early in the process of developing relationships with communities and figuring out what engagement will look like. Creating a toolbox for new centers that includes information about these first-generation teams would help make sure that these early lessons learned are not lost. For example, such a toolbox could include relevant literature and resources; background on SRP-wide conversations about engagement; and (when enough data become available) tools for assessing community needs, selecting appropriate engagement activities, and evaluating success. In order to address the clean-up gap [1], new CEC teams need to be able to build on what other teams have already done, not reinvent the engagement process each time, and this sort of guidance would help them do so.
- *Encouraging more consistent and thorough documentation of community characteristics, engagement activities, and outcomes*. As discussed previously, both communities and engagement activities vary widely, so it is all but impossible to uncover systematic patterns in what works based on the available data. To gain a better understanding of how community needs differ and, accordingly, how engagement must differ in order to be successful, much more information is needed. Because there are only a small number of SRP centers around the country, building partnerships with EPA community involvement staff, who have experiences working with a much wider range of communities, may be necessary to gather sufficient data to develop recommendations about specific engagement practices. Employing tools such as stakeholder analysis [40] and cultural humility training [41] would also help ensure that marginalized groups are included in this process and that the full range of community concerns are understood before conclusions are drawn.
- *Encouraging more reflection and research on community engagement across SRP teams*. In addition to better documentation, developing a valid set of best practices in community engagement for CEC teams requires more reflection and research that spans different centers. Although more center-specific research and reflection are also needed, general guidelines cannot be developed without a better understanding of important similarities and differences between communities. As discussed previously, experiences in different communities appear to cultivate not only different practices, but entirely different philosophies of engagement among teams, so no one team is likely to be able to offer advice that will be useful in every context. Instead, developing useful advice will require reflection and research on the activities of multiple teams in multiple contexts.

## 5. Conclusions

Overall, it is clear that there is not yet enough information to answer Dearing’s [1] call for a set of practices that can be shared, but the recommendations in the previous section provide guidelines for how to work strategically toward developing that set of practices. Initiating more conversations across the SRP about what constitutes community engagement, providing CEC teams with more guidance, working to improve documentation of engagement activities, and engaging in more research and reflection that spans different centers would help the SRP community learn from one another and make meaningful progress.
